# Biodistribution and Tumors MRI Contrast Enhancement of Magnetic Nanocubes, Nanoclusters, and Nanorods in Multiple Mice Models

**DOI:** 10.1155/2018/8264208

**Published:** 2018-09-24

**Authors:** V. Naumenko, A. Garanina, A. Nikitin, S. Vodopyanov, N. Vorobyeva, Y. Tsareva, M. Kunin, A. Ilyasov, A. Semkina, V. Chekhonin, M. Abakumov, A. Majouga

**Affiliations:** ^1^National University of Science and Technology (MISIS), Moscow 119049, Russia; ^2^M.V. Lomonosov Moscow State University, Moscow 119991, Russia; ^3^Department of Medical Nanobiotechnology, Russian National Research Medical University, Moscow 117997, Russia; ^4^D. Mendeleev University of Chemical Technology of Russia, Moscow 125047, Russia

## Abstract

Magnetic resonance imaging (MRI) is a powerful technique for tumor diagnostics. Iron oxide nanoparticles (IONPs) are safe and biocompatible tools that can be used for further enhancing MR tumor contrasting. Although numerous IONPs have been proposed as MRI contrast agents, low delivery rates to tumor site limit its application. IONPs accumulation in malignancies depends on both IONPs characteristics and tumor properties. In the current paper, three differently shaped Pluronic F-127-modified IONPs (nanocubes, nanoclusters, and nanorods) were compared side by side in three murine tumor models (4T1 breast cancer, B16 melanoma, and CT26 colon cancer). Orthotopic B16 tumors demonstrated more efficient IONPs uptake than heterotopic implants. Magnetic nanocubes (MNCb) had the highest r2-relaxivity in vitro (300 mM^−1^·s^−1^) compared with magnetic nanoclusters (MNCl, 104 mM^−1^·s^−1^) and magnetic nanorods (MNRd, 51 mM^−1^·s^−1^). As measured by atomic emission spectroscopy, MNCb also demonstrated better delivery efficiency to tumors (3.79% ID) than MNCl (2.94% ID) and MNRd (1.21% ID). Nevertheless, MNCl overperformed its counterparts in tumor imaging, providing contrast enhancement in 96% of studied malignancies, whereas MNCb and MNRd were detected by MRI in 73% and 63% of tumors, respectively. Maximum MR contrasting efficiency for MNCb and MNCl was around 6-24 hours after systemic administration, whereas for MNRd maximum contrast enhancement was found within first 30 minutes upon treatment. Presumably, MNRd poor MRI performance was due to low r2-relaxivity and rapid clearance by lungs (17.3% ID) immediately after injection. MNCb and MNCl were mainly captured by the liver and spleen without significant accumulation in the lungs, kidneys, and heart. High biocompatibility and profound accumulation in tumor tissues make MNCb and MNCl the promising platforms for MRI-based tumor diagnostics and drug delivery.

## 1. Introduction

In recent decade, as nanotechnology and materials science have progressed, nanomaterials have been mass produced and widely applied in various fields of medicine. Nanoparticles (NPs) are known to extravasate through fenestrations in abnormal neovasculature and accumulate in malignant tissues due to poor lymphatic drainage typical for tumors [1]. This phenomenon called the EPR effect is helpful in the effective delivery of the nanoparticles to the solid tumors [2]. Magnetic nanoparticles (MNP) are able to respond to and being manipulated by external magnetic fields, enabling for tumor diagnostics using magnetic resonance imaging (MRI; [3]) and more recently magnetic particle imaging (MPI; [4]). Moreover, MNP can be used for MRI-controlled drug delivery to tumors and as a prognostic tool for evaluation EPR effect in individual tumor type/patient prior to nanodrug administration [5, 6]. There are two main types of contrast agents to improve the visualization of tumors with nanoparticles. T1 MRI contrast agents shorten the spin-lattice relaxation time of neighboring protons [7], whereas T2 contrast agents improve spin-spin relaxation to reduce tissue signal [8].

Iron oxide nanoparticles (IONPs) remain among the best choice for bioimaging studies being nontoxic and biodegradable [9–11]. However, the cytotoxic effects of IONPs on the cytoskeleton of growing human neurons, melanoma cells, and umbilical vein endothelial cells (HUVEC) have been reported [12–14] suggesting that toxicological studies of IONPs should not be underestimated. Multiple IONPs have been proposed for enhancing contrast in MRI, but insufficient accumulation at tumor bed and low magnetic performance hamper the translation into clinics. Thus, an ideal MNP-based MRI contrast agent should meet several requirements: (i) high delivery efficiency to target tissues, (ii) enhanced MRI contrasting properties, and (iii) high biocompatibility and low toxicity. There are several characteristics (e.g., size, shape, and surface coating) that can be effectively tuned to control both biological and magnetic performance of potential contrast agents.

Core size (dC) of the iron oxide nanoparticles plays a very important role in determining its saturation magnetization and dictates relationship between T1 and T2 relaxation times when used as MRI contrast agents [7, 11]. Thus, increasing dC of IONPs from 5 to 14 nm results in 3-log increased T2-relaxivity [15]. Hydrodynamic size (hD) of the IONPs is one of the most important factors that determines biodistribution kinetics [16]. IONPs with hD < 10 nm are rapidly filtered by the kidneys, whereas those with hD > 100 nm are mostly entrapped by the liver and spleen [11]. As a result, NPs with hD < 100 nm tend to show a higher delivery efficiency than larger particles [17]. Therefore, by tuning the hD of the IONPs between 10 and 100 nm, it is possible to extend the blood half-life and increase the access of the IONPs to tumors [18]. As larger core sizes result in larger hD, for the best MRI performance a trade-off should be found between higher imaging efficiency and longer blood residence time [11]. From safety prospective, NPs below 5 nm diameter are the most hazardous due to possible nuclear penetration and very high surface area over volume ratios [19].

Nanostructures with a high length to width aspect ratio (gold nanorods and iron oxide “nanoworms”) have shown longer blood circulation times over the spherical counterparts [20, 21]. The possible mechanism is attributed to lesser uptake by macrophages due to an opsonin-independent phagocytosis phenomenon [20]. Organs biodistribution of nanorods and nanospheres also differed with preferential accumulation in lymph nodes for the former and the highest uptake by the liver/spleen for the latter [22–24]. Presumably due to longer half-life rod-shaped nanoparticles exhibit a higher delivery efficiency to malignant tissues compared with spherical, plate or flake, and other shapes, with values of 1.1%, 0.7%, 0.6%, and 0.9% injected dose (ID), respectively [17]. On the cellular level for Qdots [25] and gold nanoparticles [26] with higher aspect ratios lower internalization efficiency was described accompanied with lower cytotoxicity. Other NP morphologies (such as cubes or clusters) are less studied [27], and effects of these specific shapes on its pharmacokinetics and biodistribution mechanisms are still unknown.

Nanoparticles magnetic performance is also shape dependent. Thus, colloidal nanocrystal clusters have been suggested as perspective nanoplatform for bioimaging [28]. Magnetic nanocrystals assembling in secondary structures results in an enhanced saturation magnetization compared with that of individual nanocrystals, and a stronger perturbating local magnetic field in its vicinity can be expected [29]. Recent studies have shown that the transverse relaxivity (*r*2) is 3- to 5-fold higher compared with individual nanocrystals [9, 30] and commercial products, such as the Endorem [10, 28]. Moreover, it has been reported that longitudinal relaxivity (*r*1) is also enhanced in multicore nanoparticles [9, 31]. Despite promising results on nanoclusters *r*1 and *r*2 properties, there are only few works focusing on its MRI contrasting properties for tumor diagnostics [32]. Since most of the current diagnostic approaches are based upon spherical nanostructures, understanding the implications of other shapes will allow for the development of improved tumor imaging [33].

Uncoated IONPs are often colloidally unstable and get rapidly eliminated by the macrophages. Different types of natural and synthetic polymers have been used to improve the pharmacokinetic performance of the IONPs [11]. Pluronic F-127 is a perspective surface coating allowing nanoparticles to have higher aggregate stability [34] and biocompatibility [35].

Apart from nanoparticles' properties, biological factors also determine IONPs pharmacokinetics and biodistribution. Variations in the tumor-targeted delivery of the IONPs through the EPR effect have been attributed to differences in animal models, mice strain, and biological diversity of the tumors [36, 37]. Although more and more data are becoming available regarding size and shape impact on IONPs performance in MRI, most of data are derived from stand-alone studies, where a single type of IONPs with specific physicochemical properties is delivered to a certain tumor type under arbitrarily selected conditions. In the current paper, we describe MRI-contrast properties of Pluronic F-127-covered magnetic nanoclusters (MNCl), nanocubes (MNCb), and nanorods (MNRd) in mouse breast cancer (4T1), colon cancer (CT26), and melanoma (B16) models. Based on delivery efficiency to malignant tissues, magnetic properties, and safety studies, we show that MNCb and MNCl are effective contrast agents and promising vehicles for drug delivery to different tumor types.

## 2. Materials and Methods

### 2.1. Synthesis of IONPs

MNCb and MNCl were synthesized as previously described [38, 39]. MNRd were synthesized by two-step method. *β*-FeOOH nanorods were obtained by hydrolysis of FeCl_3_ water solution in the presence of brunched high-weight polyethyleneimine (PEI, *M*_w_ = 25000). First, 2 mL of PEI was dissolved in 100 mL of deionized water and then 2.54 g of FeCl_3_ was added to the obtained solution. The mixture was heated at 80°C under magnetic stirring for 2 h. After cooling the mixture to room temperature, the pH was adjusted to 7.0 by adding 2 M NaOH water solution. The precipitate was separated by centrifugation and washed several times with deionized water. 15 mL of *β*-FeOOH nanorods with iron concentration 3 mg/mL was mixed with 50 *µ*L of hydrazine hydrate. The mixture was placed in hermetic ampoule, which was then undergone by 3 microwave irradiation cycles (each cycle included heating solution to 100°C, sustaining for 30 s and cooling down to 35°C). The final product was separated from the solution by permanent magnet and washed with deionized water several times. 1 mL of MNRd was mixed with 4 mL of Pluronic F-127 water solution (4 mg/mL). The obtained solution was stirred by vortex for 1 h at room temperature. The final product was centrifuged using 100 kDa centrifugal tubes and washed 2 times with deionized water to purify MNRd from unbound Pluronic F-127.

### 2.2. Characterization of IONPs by Transmission Electron Microscopy (TEM)

TEM images of IONPs were taken on JEOL JEM-1400 (120 kV) microscope. All samples were prepared by dropping a water dispersion of synthesized samples onto a carbon-coated copper grid (300 mesh) and subsequent evaporating of the solvent. The average diameter of the samples and size distribution were evaluated using ImageJ software. At least 1000 IONPs were analyzed for each sample.

### 2.3. Characterization of IONPs by MRI

The T2-relaxation rate of water protons in the presence of IONPs covered with Pluronic F-127 was measured in 500 *μ*L test tubes at 18°C on a ClinScan 7T MRI system. Image acquisition was performed in Spin Echo mode with the following parameters: MRI system TR = 10,000 ms, TE = 8, 16, 24, and 240 ms, ﬂip angle = 180°, resolution 640 × 448 pixels, and ﬁeld of view (FOV) = 120 × 82.5 mm^2^. Signal intensities from regions of interest were manually measured by ImageJ software, and the T2-relaxation time was calculated by ﬁtting the signal from images with different TE. T2-relaxivity values were calculated using a linear ﬁtting of 1/T2 relaxation times to iron concentration. The slope of the ﬁtting curve represents the R2-value for IONPs covered with Pluronic F-127 used for MR imaging.

### 2.4. Dynamic Light Scattering (DLS) and Zeta-Potential Measurements

Hydrodynamic size and zeta-potential of IONPs were determined by Malvern Zetasizer Nano ZS. The concentration of IONPs water solution was 0.5 mg/mL for each sample.

### 2.5. Cells

All cell lines were purchased from the American Type Culture Collection (ATCC, Manassas, VA, USA). 4T1, mouse breast cancer cells, and CT26, mouse colon carcinoma cells, were cultured in RPMI-1640 medium (Gibco). B16-F10, mouse melanoma cells, were cultured in DMEM medium with 4.5 g/L glucose (Gibco) and SC-1, feral mouse embryo cells-in DMEM with 1 g/L glucose (Gibco). All culture media were supplemented with 10% FBS and 2 mM L-glutamine (Gibco), and for CT26 cell line, 10 mM HEPES (Helicon) and 1 mM sodium pyruvate (Gibco) were added. Cells were maintained at 37°C in a humidified incubator supplied with 5% CO_2_.

### 2.6. MTS Assay

SC-1 cells were plated at concentration of 25,000 cells per well in 96-well plates. After 48 h, IONPs were added to the cells at final concentration 1–200 *µ*g/mL. PBS and DMSO (25%) served as negative and positive controls, respectively. After 48 h incubation with IONPs, cells were washed with PBS, and 20 *μ*L of MTS reagent (CellTiter 96 AQueous Non-Radioactive Cell Proliferation Assay, Promega, USA) was added to each well with 100 *μ*L of culture medium. After 4 h incubation at 37°C in darkness, 100 *μ*L of culture medium with MTS from each well was carefully replaced in new plates to avoid the presence of nanoparticles in the analyzed solution. The absorbance of the obtained solution was measured at 490 nm using Thermo Scientific Multiskan GO spectrometer. Experiments were performed in triplicates.

### 2.7. ROS Detection by 2′,7′-Dichlorodihydrofluorescein Diacetate (H2DCFDA)

Cells were plated in the wells of Stripwell 96-well plates (Corning) at concentration of 25,000 cells per well and cultured at 37°C in a humidified incubator. After 48 h IONPs (200 *µ*g/mL iron) were added to cells for 6 or 24 h. Cells incubated in culture medium or in medium with PBS were used as controls. To detect ROS in cells after incubation with IONPs, unfixed cells were washed with HBSS (Gibco), supplemented with 2 mM L-glutamine and 10 mM HEPES (Helicon) (pH 7,4 adjusted with 1 N NaOH), and stained with 2 *µ*M H2DCFDA solution (Life technologies) in HBSS for 30 min at 37°C in darkness. Then, cells were carefully washed with HBSS 3 times for 5 min. The obtained samples were analyzed at fluorescent microscope EVOS (Life technologies), objective PlanFluor 20x/0.45. Experiments were performed in duplicates.

### 2.8. Apoptosis/Necrosis Detection

Cells were plated in the wells of Stripwell 96-well plates. After 48 h, IONPs (200 *µ*g/mL iron) were added to cells for 6 and 24 h. Cells incubated in free culture medium or in medium with PBS were used as controls. Cells were washed with HBSS supplemented with L-glutamine and HEPES and intravitaly stained with DNA Nuclear Green DCS1 dye (Abcam) for 40 min at room temperature in the darkness, and washed twice with HBSS. The photos were captured by fluorescent microscope EVOS (objective PlanFluor 20x/0.45) and analyzed in ImageJ software. Experiments were performed in duplicates.

### 2.9. Animals and Tumor Models

All animal experiments were approved by N.I. Pirogov Russian National Research Medical University bioethical committee (protocol ## 25/2017, 26/2017). Six- to eight-week-old female BALB/c and C57BL/6 mice were obtained from Andreevka Animal Center (Andreevka, Russia). At the time of use, animals were between 7 and 11 weeks old and weighed 20–22 g. 4T1, CT26, and B16 tumors were established by subcutaneous injection into the hind flanks of 1 × 10^6^, 1.5 × 10^6^, and 5 × 10^6^ cells, respectively. When tumors reached ∼40 mm^2^ (8 to 12 days after cell implantation), 5 mg/kg IONPs were intravenously (i.v.) injected.

### 2.10. MRI

For in vivo studies, images were obtained using a 20-cm volumetric coil as a transmitter and a 4-segment surface coil as a receiver of the RF signal. Tumor-bearing mice were anaesthetized with 2% isoflurane and scanned before and 1 h, 6 h, and 24 h after i.v. injection of 5 mg/kg MNCb, MNRd, and MNCl (*n*=5 for each group). The following regimens were used: (a) fat-suppressed T_2_-weighted turbo spin-echo (TSE) (TR = 2000 ms, TE = 42 ms, FOV = 60 × 35.625 mm, base resolution (640 × 380)) and (b) *T*_2_^*∗*^-weighted gradient echo (GRE) (TR = 400 ms, TE = 3.46 ms, FOV = 31.25 × 40 mm, base resolution (200 × 256)). Images were processed in RadiAnt DICOM Viewer.

### 2.11. Biodistribution Studies by AES

Tumor-free mice were injected with 5 mg/kg IONPs and sacrificed 1 h or 24 h after injection (*n*=5 for each group) by cardiac perfusion with 30 mL PBS under anesthesia. Liver, spleen, kidneys, lungs, and heart were collected, weighted, and digested in aqua regia during 24 h. Quantification of the iron concentration was carried out by atomic emission spectroscopy (Agilent 4200 MP-AES, USA) using the calibration curve for the standard samples in 0.1-1 mg/mL concentration range. Untreated animals (*n*=5) were used as control for measuring endogenous iron levels. Mean iron levels in control organs were subtracted from corresponding iron levels in MNCb-, MNRd-, and MNCl-treated groups to get IONPs-associated iron concentration (*µ*g/g tissue). IONPs delivery efficiency calculations were based on iron concentration in the tissues, organ mass, and injected dose. For tumor-bearing animals, iron concentration in organs and tumors was measured in untreated and IONPs-treated (5 mg/kg) groups 24 h upon i.v. injection as described above.

### 2.12. Statistical Analysis

Plotting and calculation of the standard deviation (SD) and standard error of mean (SEM) values were made in GraphPad Prism 5. Data were analyzed using the analysis of variance (ANOVA) test, *χ*-square test, unpaired *t*-test, and Dunnett's multiple comparison test. *p* values <0.05 were considered significant.

## 3. Results

### 3.1. Synthesis and Characterization of IONPs

**Figure 1:**
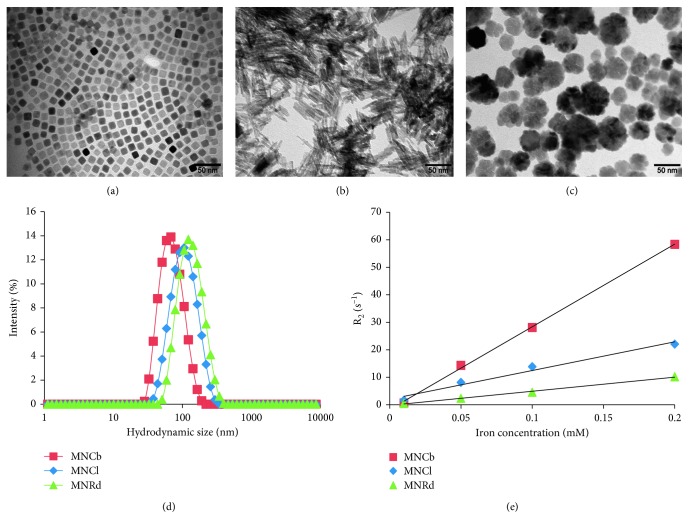
Physicochemical properties of magnetite nanoparticles (a–c). TEM image of MNCb (a), MNRd (b), and MNCl (c). (d) DLS measurement after conjugation of IONPs with Pluronic F-127. (e) Plot of r_2_ values of IONPs.

The synthesis and detailed physicochemical characterization of Pluronic F-127-modified MNCb and MNCl have been previously described [38, 39]. Data on core/hydrodynamic size, relaxation rate, and surface charge of studied IONPs are summarized in Figure 1, Figure S1, and Table 1.

All three IONPs had a comparable crystallite size and surface charge. As expected, MNCl core diameter was larger than those of MNCb and MNRd and the differences between hD were even more profound due to IONPs geometry. Magnetic measurements showed that MNCl had the highest value of saturation magnetization (80.5 emu/g; Figure S2) compared with MNRd and MNCb (54.4 and 48 emu/g, respectively). On the contrary, MNRd demonstrated the highest value of coercive field, 141 Oe, that was 3- and 9-fold higher than the corresponding values for MNCl and MNCb (Figure S2).

The XRD measurements of MNCb, MNRd, and MNCl showed that all samples can be attributed to magnetite phase Fe_3_O_4_ (*a* = 8.396 Å, ICDD no. 19–0629). The XRD spectra and characteristics of the obtained samples are presented in Figure S3 and Table S1, respectively.

### 3.2. Cytotoxicity Studies

**Figure 2:**
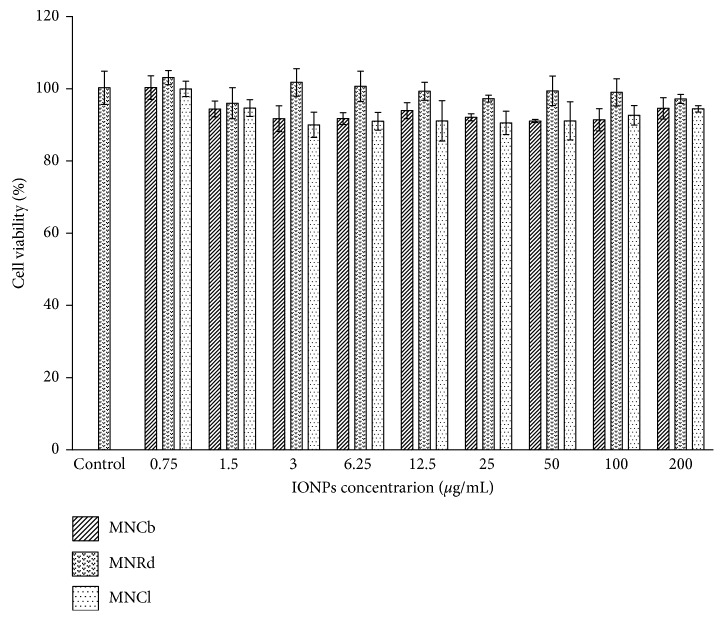
IONPs in vitro toxicity. SC-1 cells viability assessed by MTS-test after 48 h co-incubation in culture medium with PBS (control) or IONPs in concentration range of 0.75–200 *µ*g/mL. Results are shown as means ± SD.

IONPs cytotoxicity was studied on normal mouse fibroblasts. All samples were not toxic in MTS test up to 200 *µ*g/mL concentration (Figure 2), which is equivalent to the calculated plasma IONPs levels after a typical i.v. injection of 5-10 mg/kg in rodents.

To further confirm IONPs biocompatibility cells were stained with H2DCFDA and nuclear green after exposure to the highest concentration of MNCb, MNRd, and MNCl (200 *µ*g/mL). Consistent with MTS results, no difference in ROS production (Figure S4) and cell death marker (Figure S5) was found between IONPs-treated and control cells after 6 and 24 h co-incubation. Overall, at the evaluated IOPNs concentration, no effect on cell viability, ROS production, or cell damage was observed.

### 3.3. IONPs Biodistribution

**Figure 3:**
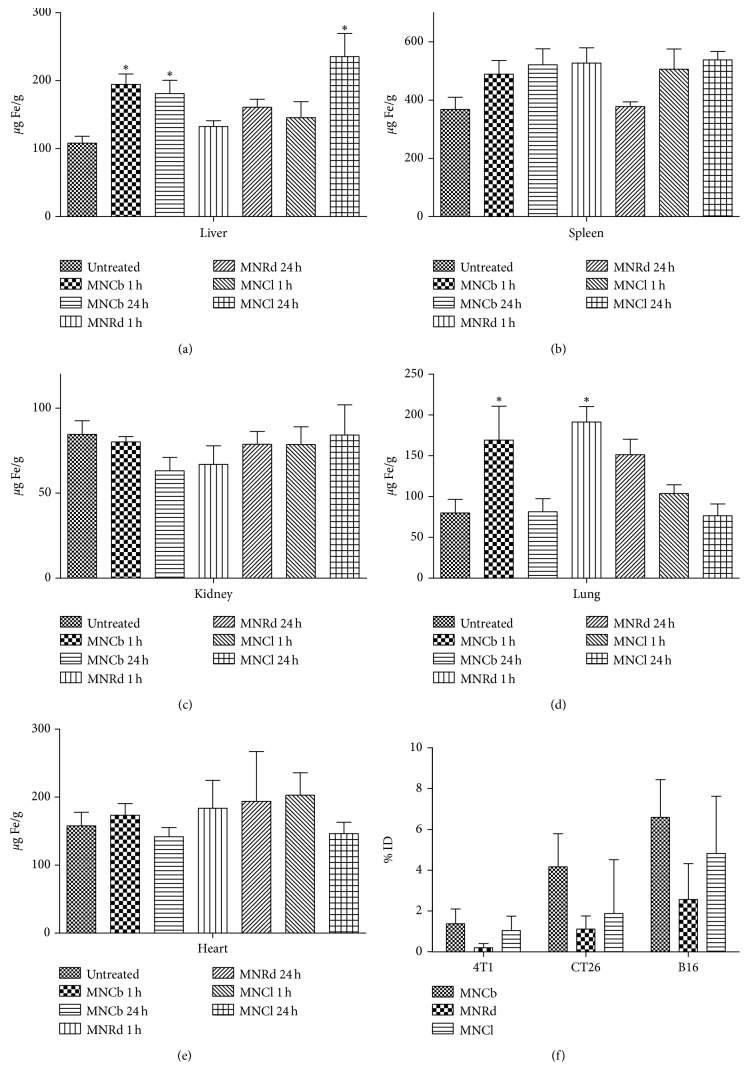
IONPs biodistribution (a–e). Iron levels measured by AES in liver (a), spleen (b), kidney (c), lung (d), and heart (e) in untreated and IONPs-treated tumor-free animals 1 and 24 h after i.v. injection (5 mg/kg). (f). Percentage of injected dose delivered to 4T1, CT26, and B16 tumors 24 h after IONPs systemic administration. Results are shown as means ± SEM. ^*∗*^ *p* < 0.05 in IONPs-treated vs control group (Dunnett's multiple comparison test).

Iron concentrations in the liver, spleen, kidneys, lungs, and heart were measured by AES in nontreated and IONPs-treated (5 mg/kg) tumor-free animals (Figures 3(a)–3(e)). One hour after systemic injection, MNCb were mostly captured by liver, followed by lungs, although after 24 h, iron concentration in lungs decreased back to the normal level. MNRd accumulated in lungs starting from early time point, whereas liver capturing was lower than for MNCb. In MNCl-treated mice, iron level in liver increased only 24 h after i.v. injection, whereas lung iron concentration did not differ from control animals. For all studied IONPs, iron concentrations in spleen were slightly higher (although nonsignificant) than in nontreated controls, and none of the particles accumulated in the kidneys and heart.

Tumors are known to affect organs vascularization, morphology, and function through cancer cells dissemination and/or stimulating myeloid-derived suppressor cells maturation and recruitment. For instance, spleen weights were significantly higher for 4T1-bearing animals when comparing with controls and animals with CT26 tumor implants (251 ± 19 mg, 157 ± 7 mg and 144 ± 4 mg, respectively). Therefore, IONPs biodistribution may differ between tumor-free and tumor-bearing animals. To check it, iron concentration was measured in mice with implanted 4T1, CT26, and B16 tumors 24 h after IONPs injection (Figure S6). Qualitatively the same results were obtained for tumor-bearing animals identifying liver and spleen as primary accumulation site for studied IONPs. Of note, MNRd accumulation in lungs was a consistent finding in all animal groups. No difference was found between BALB/c and C57BL/6 mice in IONPs biodistribution profiles.

It is known that nanoparticles accumulate in solid tumors in EPR-dependent manner. To address this point, iron levels were measured in 4T1, CT26, and B16 tumors 24 h after IONPs injection (Figure 3(f)). Indeed, IONPs accumulation in malignancies was dependent on tumor type (two-way ANOVA, *p*=0.12) with maximum IONPs delivery rate in B16 tumors (4.1% ID) and minimum in 4T1 tumors (0.8%ID) (unpaired *t*-test, *p*=0.036). MNCb, MNRd, and MNCl delivery efficiency for all tumor types was 3.79% ID, 2.94% ID, and 1.21% ID, respectively (two-way ANOVA, *p* > 0.05).

### 3.4. MRI

Next MRI studies were performed to evaluate MNCb, MNRd, and MNCl contrast properties in vivo. 4T1, CT26, and B16 tumors were imaged before and after IONPs systemic injection (5 mg/kg). Each animal was scanned using *T*_2_ regimen, and then tumors were imaged with *T*_2_^*∗*^ settings (Figure S7).

IONPs performance in MRI was assessed by percentage of tumors with enhanced contrast (Table 2) and type of signaling (focal or diffuse contrasting, Figure S7). Previously it has been shown that some nanoparticles (nanospheres and nanodisks) mainly delivered to tumor border, whereas others (nanorods and nanocages) demonstrated even accumulation throughout the tumors [40]. Local and diffuse MR-contrasting can be attributed to differences in IONPs distribution within tumors.

**Figure 4:**
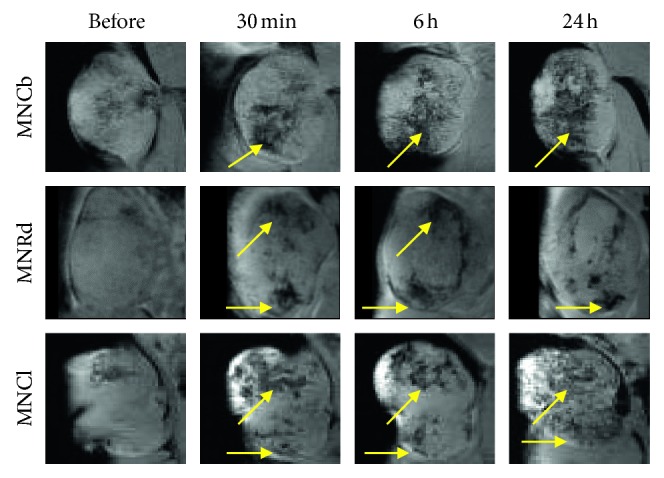
Dynamics of MNPs accumulation in B16 tumors. Representative *T*_2_^*∗*^-weighted MR images of B16 tumors captured before and within 24 h after i.v. injection of IONPs (5 mg/kg). Foci of enhanced tumor contrasting are shown by arrows.

**Figure 5 fig5:**
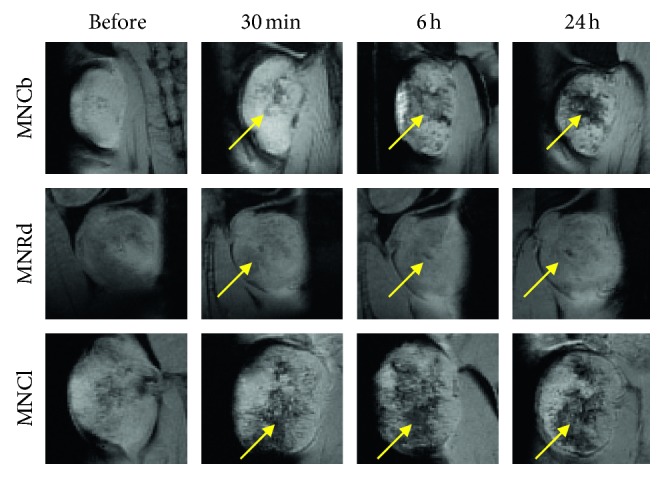
Dynamics of IONPs accumulation in 4T1 tumors. Representative *T*_2_^*∗*^-weighted MR images of 4T1 tumors captured before and within 24 h after i.v. injection of IONPs (5 mg/kg). Foci of enhanced tumor contrasting are shown by arrows.

**Figure 6:**
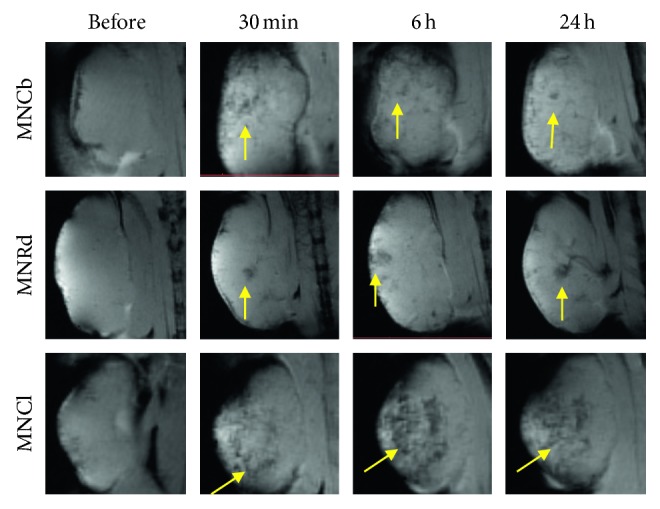
Dynamics of MNPs accumulation in CT26 tumors. Representative *T*_2_^*∗*^-weighted MR images of CT26 tumors captured before and within 24 h after i.v. injection of IONPs (5 mg/kg). Foci of enhanced tumor contrasting are shown by arrows.

Consistent with biodistribution data, the best tumor enhancing contrast was revealed in B16 model. IONPs were detected in 88% of the tumors, primarily by diffuse signal decrease in tumor tissues (Table 2 and Figure 4). 4T1 tumors imaging with enhanced IONPs contrasting was also effective (82% for three IONPs), while signaling (mainly focal) from CT26 was detected by MRI only in 63% of tumors (Table 2 and Figures 5 and 6).

Among studied IONPs, MNCl demonstrated the best MRI-contrast properties (Table 2). Diffuse contrasting was found in 96% of the tumors after MNCl administration (Figures 4 [Fig fig5]–6 lower panels). MNCb and MNRd provided detectable tumor signaling in 73% and 63% tumors, respectively (Figures 4 [Fig fig5]–6). Interestingly, MNCb provided better contrasting for 4T1 tumors, but were not effective in B16 tumors (100% and 57% tumors, respectively), whereas the opposite trend was observed for MNRd, enabling enhanced contrast for 60% 4T1 and 100% B16 tumors.

MRI was used to track the dynamics of IONPs accumulation in malignant tissues and to identify the optimal time point for tumor visualization. The most profound tumor contrasting corresponded to 6-24 hours after MNCb and MNCl injection, whereas MNRd accumulation in malignancies reached its maximum within first 30 minutes upon i.v. administration (Figures 4 [Fig fig5]–6). Among tumor models, CT26 maximum uptake for MNCb and MNRd was around 6 hours after treatment (Figure 6), but 4T1 and B16 tumors kept accumulating IONPs up to 24 h after injection (Figures 4–5).

## 4. Discussion

Although extensive work yielded many valuable insights on the contributions of particle size and surface chemistries to in vivo tumor imaging, information on the biological influence exerted by particle shape is relatively lacking [33]. Visualization efficiency is dependent on both IONPs delivery to targeted tissues and magnetic performance. In the current study, three IONPs were tested in vitro and in vivo to characterize its biocompatibility, biodistribution, and contrast properties. The rationale of studying given shapes, sizes, and structures was based on previous biodistibution and imaging data. Thus, nanocubes (along with nanospheres) are currently the most commonly used contrast agents demonstrating reasonable contrast enhancement. However, there is growing evidence that these nanostructures are less efficient in delivery to malignant tissues than nanorods [17, 21, 24]. Also, while nanospheres and nanodiscs are only observed on tumor edges, nanorods were redistributed throughout the tumors [40]. Due to its dual nature, nanoclusters are of special interest for MRI diagnostics. A cluster can be considered as an individual nanoobject, but its magnetic performance is determined by the contribution of each nanoparticle. As a result, MNCl can exhibit increased values of saturation magnetization and relaxivity compared with individual nanoparticles [9, 10, 30].

Despite numerous studies on IONPs in vivo imaging, only few of them compare particles under the same conditions [21, 24]. Most data regarding size or shape impact on bioperformance are coming from a side along studies with incomparable tumor models, treatment schedules, and variable IONPs parameters. To check if shape and structure determine MRI contrasting, we aimed to keep other IONPs physicochemical properties consistent. Thus, all IONPs were coated with Pluronic F-127 and had similar values of surface charge. Pluronics are made up of poly(ethylene oxide)-poly(propylene oxide)-poly(ethylene oxide) (PEO-PPO-PEO), in which PPO is the hydrophobic segment and contributes for 30% of the block copolymer, whereas PEO is a hydrophilic segment contributing 70% of the block copolymer [41]. Previous studies have demonstrated that Pluronic F-127 has an advantage over other pluronics in its ability to redistribute IONPs to tumor tissues [34]. All studied IONPs had a similar crystallite size of 10–20 nm; however, hD ranged from 65 nm for MNCb to 120 nm for MNRd. It should be noted that maintaining all the other parameters effectively the same and only change the core morphology is impracticable for such different shapes as nanocube and nanorod [11]. Nevertheless, all studied IONPs had hD preferable for evading macrophage capturing and renal elimination and entering the tumors by EPR [42–44].

Among the three IONPs, MNCb exhibited the highest relaxivity rates in vitro and the best delivery efficiency to tumor site. However, MNCl overperformed MNCb in contrasting malignant tissues. Discrepancy between in vitro and in vivo *T*2-relaxivity estimation is probably due to different degrees of freedom that a tissue can impose on the MRI signal. For instance, distribution profiles throughout the tissues (deep penetration or superficial accumulation) may affect intensity and type of signaling (diffuse or local, respectively). Nanorods had the lowest *r*2-relaxivity, the less efficient accumulation in tumors that resulted in poor MRI performance in tumors.

Despite some variability between mice models, the overall results clearly indicate that the liver and spleen captured the majority of IONPs consistent with multiple previous reports [11, 16, 24, 45]. Thus, 24 h after treatment with MNCb, MNRd, and MNCl, liver accumulated 74 ± 12% ID, 50 ± 9% ID, and 70 ± 6% ID and spleen 20 ± 3% ID, 11 ± 3% ID, and 21 ± 4% ID, respectively. The lower MNRd uptake rate by the liver and spleen is probably due to elongated nanoparticles' lower sequestration by macrophages in comparison to other shaped counterparts [33]. Opposite to previous report, MNCl did not accumulate neither in kidneys [45] nor in heart and lungs. In some mice models, we found MNCb in these organs; however, it was not a consistent finding. Meanwhile, significant amount of MNRd were found in lungs,17.1% ID, in all studied models that is comparable to the spleen capturing rate. There are a few reports showing the presence of a smaller fraction of i.v. injected IONPs in lungs [46, 47]. Also 9 nm MNP modified with dimercaptosuccinic acid (DMSA) targeted preferentially to the lung after i.v. administration [48, 49]. Authors suggested that this specific targeting was due to surface chemistry, but the exact mechanism remains unclear. ICAM-1-directed submicron-sized spheres and elliptical disks also effectively targeted lung endothelial cells [50]. In the current study IONPs were not functionalized by specific ligands and MNRd accumulation in lung capillaries may be attributed to nanorods tumbling behavior under flow, resulting in decreased floating speed and margination towards the vessel wall, and thus increasing chances for MNRd adhesion to endothelial cells [50]. As lung is the first filter for i.v. injected particles, it is natural that significant part of MNRd settled down in lung capillaries. MNRd accumulation in lungs can be used for lung imaging or delivering drugs to lung primary tumors or metastases. Although the previous study in rodents has shown that the clearance rate of the nanoparticles is dependent on the mice strain type [36], we have not found any significant differences in biodistribution profiles neither between BALB/c and C57BL/6 mice nor in healthy and tumor-bearing animals.

IONPs accumulation via EPR effect varies greatly among tumors depending on the location, size, and structure/histology. Thus, rapid-growing tumors have more leaky vessels contributing to NPs enhanced extravasation. The ratio between tumor parenchyma and stroma determines tumor tissue consistency and tension, therefore affecting lymphatic drainage [1]. Using several tumor models is highly favorable for comprehensive analysis of IONPs bioperformance. In the current study for the first time IONPs uptake was estimated in three different mice tumor models. Both MRI and AES identified maximum IONPs accumulation in B16 tumors. It is known that orthotopic tumors tend to accumulate NPs more efficiently than heterotopic counterparts [17]. Most likely, subcutaneous implantation provides natural microenvironment for melanoma cells resulting in higher uptake rates compared with heterotopic implants of colon cancer and breast cancer cells.

Tumor accumulation dynamics varies significantly for different nanoparticles. Thus, the highest uptake for gold nanospheres and nanocages was observed at 24 hours after i.v. administration, while nanodisks reached accumulation peak in tumor tissues at 6 hours after injection [40]. Studying IONPs delivery to tumor tissues is crucial for identifying an optimal time point for MRI-based tumor diagnostics. Probably, due to increased half-lives, MNCb and MNCl gradually accumulated in most tumors up to 24 h, resulting in better uptake rates and MRI performance compared with MNRd. The latter demonstrated peak accumulation rates within first 30 minutes after injection without significant increase at later time points. Previously it has been shown that elongated nanoparticles are more effective at evading nonspecific uptake by Kupffer cells in comparison to other shaped nanoparticles displaying prolonged blood circulation and distribution to specific organs or tumor sites [33]. Indeed, in the current study MNRd accumulation in the spleen and liver was lower than that for other IONPs, but the particles targeted the lungs rather than tumors. MNRd rapid clearance is the most likely explanation of MNRd low delivery efficiency and poor MR contrasting. Also, nanorods may have shorter circulation half-lives due to the lower coverage density of coating molecules on its surface [40].

Delivery efficiency to tumor site is a key parameter for IONPs-based cancer diagnostics and therapy. According to recent comprehensive review on nanoparticles delivery, only around 0.7% ID accumulates in tumor after systemic injection [17]. In the current work, 2.94% of injected MNCl and 3.79% of injected MNCb ended up in studied tumors, and for B16 melanoma, uptake rates were even higher (4.8% and 6.6% ID, respectively). These values are much better than average nanoparticle delivery efficiency, although at least four groups got more than 5% ID in the tumors [17]. The results suggest that described IONPs are promising not only for diagnostics but also for targeted drug delivery. It should be noted that MNCb hD was lower than that of MNCl and MNRd (see Table 1), so it cannot be ruled out that better MNCb uptake is not only a shape- but also size-dependent phenomenon.

It is commonly accepted that IONPs are highly biocompatible, which makes them attractive for medical applications [11, 51, 52]. To ensure the safety of studied MNCb, MNRd, and MNCl, we carried on cytotoxicity studies on mice fibroblasts. Normal cells are preferential for in vitro tests as cancer cells have several specific characteristics making them less sensitive to some NP-mediated toxic effects. In vitro tests did not show any toxicity of studied IONPs at concentrations up to 200 *µ*g/mL in agreement with numerous reports [9, 10, 45, 53]. For tumor imaging, we used a standard dose of 5 mg/kg that corresponds to plasma levels of 100 *µ*g/mL in 20 g mice. This dose was well tolerated consistent with previous studies where i.v. injection of 5, 10, 20 and even 100 mg/kg iron was found safe in rodents [16, 35, 51, 52, 54]. In early toxicological study, no acute toxicity was observed in animal experiments at concentrations up to 168 mg Fe/kg [55]. Biodistribution studies demonstrated that the injected dose used for MRI is lower than the total body iron content. Thus, 24 h after injection in BALB/c mice, iron concentration in liver, that mainly accumulates IONPs, increased from 137 ± 17 to 229 ± 17, 192 ± 17, and 238 ± 17 *µ*g/g for MNCb, MNRd, and MNCl, respectively. These concentrations are significantly lower than the level required for cirrhosis and/or hepatocellular carcinoma development (4000 *µ*g/g). Collectively, the data suggest that studied IONPs are biocompatible and safe for biomedical purposes.

## 5. Conclusions

All studied IONPs were found safe and biocompatible for in vivo applications. MNCb and MNCl high *r*2-relaxivity and delivery to malignant tissues resulted in efficient MR contrasting of multiple tumors in mice. In contrast, MNRd low in vitro *r*2-relaxivity coupled high accumulation in lungs and suboptimal uptake by tumors limited its contrasting efficiency. Overall, the results suggest that synthetized MNCb and MNCl are promising for cancer diagnostics and drug delivery.

## Figures and Tables

**Table 1:** Physicochemical characteristic of IONPs.

Type of IONPs	Core size ± SD (nm)	Hydrodynamic size ± SD (nm)	*T*_2_-relaxivity (mM^−1^·s^−1^)	Surface zeta potential (mV)
MNCb	15 ± 2	65.4 ± 31.6	300	−18.5
MNRd	20 ± 7 (length), 3 ± 1 (diameter)	120.4 ± 71.1	51	−19.0
MNCl	40 ± 10	100.0 ± 42.3	104	−12.0

**Table 2:** Percentage of tumors with enhanced MR contrasting after IONPs injection.

	4T1	B16	CT26	All tumors
MNCb (%)	100	57 ± 20	70 ± 15	73 ± 10^*∗*^
MNRd (%)	60 ± 16	100	30 ± 15	63 ± 9^#^
MNCl (%)	100	100	90 ± 10	96 ± 4^*∗*^^#^
All IONPs (%)	82 ± 8	88 ± 7^*∗∗*^	63 ± 9^*∗∗*^	78 ± 5

Results are shown as mean ± SEM; ^*∗*^ *p*=0.02; ^*∗∗*^ *p*=0.003; ^#^ *p*=0.037 (*χ*-square test).

## Data Availability

The data used to support the findings of this study are available from the corresponding author upon request.

## Supplementary Materials

Supplementary Materials Supplementary figures S1–S7 and Table S1 can be found in the online version of this article at the publisher's website. Supplementary figure S1. Histograms of MNCb (a), MNRd (b), and MNCl (c) size distribution (associated with Figure 1A) Supplementary figure S2. M-H curves of MNCb, MNRd, and MNCl at room temperature. M-H hysteresis loops (from −20 to 20 kOe, 300 K) were obtained on (Quantum Design) Physical Property Measurement System (PPMS) equipped with vibration magnetometric device (VSM) with 2 mm amplitude of oscillations, 40 Hz frequency. Supplementary figure S3. X-ray diffraction patterns of MNCb, MNRd, and MNCl at room temperature. XRD patterns were obtained using an X-ray power diffractometer Rigaku Ultima IV with Co K*α* radiation at room temperature. The data were collected from 2*θ* = 20 to 80° at a scan rate 0.1° per step and 3 s per point. Supplementary figure S4. ROS detection in IONPs-treated cells. SC-1 cells intravital staining with H2DCFDA after 6 and 24 h incubation in culture medium with PBS (control) or 200 *μ*g/mL IONPs, phase contrast, and fluorescent microscopy. White arrows indicate single cells with increased level of ROS production. Supplementary figure S5. Detection of late apoptotic/necrotic cells after IONPs treatment. SC-1 cells intravital staining with Nuclear Green after 6 and 24 h incubation in culture medium with PBS (control) or 200 *μ*g/mL IONPs, phase contrast, and fluorescent microscopy. White arrows indicate rare dead cells. Supplementary figure S6. IONPs biodistribution profiles in multiple tumor models. Iron concentrations measured by AES in liver, spleen, kidney, lung, and heart of mice with 4T1 (a), CT26 (b), and B16 (c) tumors. Results are shown as means ± SEM. ^*∗*^ *p* < 0.05 in IONP-treated vs control group (Dunnett's Multiple Comparison Test). Supplementary figure S7. Patterns of MR contrast enhancing. T2-weighted imaging mode (left panels) was followed by *T*_2_^*∗*^-weighted imaging (right panels). Representative images of diffuse (A, arrow) and focal (B, arrowhead) tumor contrast enhancement after intravenous injection of IONPs. Supplementary Table S1. XRD characteristics of MNCb, MNRd, and MNCl and standard magnetite nanoparticles.
