# Supplementary material for: Biodistribution and Tumors MRI Contrast Enhancement of Magnetic Nanocubes, Nanoclusters, and Nanorods in Multiple Mice Models
Source: Contrast Media Mol Imaging. 2018 Sep 24;2018:8264208. doi: 10.1155/2018/8264208 (PMC6174815; doi:10.1155/2018/8264208)
Supplement: Supplementary Materials — Supplementary figures S1–S7 and Table S1 can be found in the online version of this article at the publisher's website. Supplementary figure S1. Histograms of MNCb (a), MNRd (b), and MNCl (c) size distribution (associated with Figure 1A) Supplementary figure S2. M-H curves of MNCb, MNRd, and MNCl at room temperature. M-H hysteresis loops (from −20 to 20 kOe, 300 K) were obtained on (Quantum Design) Physical Property Measurement System (PPMS) equipped with vibration magnetometric device (VSM) with 2 mm amplitude of oscillations, 40 Hz frequency. Supplementary figure S3. X-ray diffraction patterns of MNCb, MNRd, and MNCl at room temperature. XRD patterns were obtained using an X-ray power diffractometer Rigaku Ultima IV with Co Kα radiation at room temperature. The data were collected from 2θ = 20 to 80° at a scan rate 0.1° per step and 3 s per point. Supplementary figure S4. ROS detection in IONPs-treated cells. SC-1 cells intravital staining with H2DCFDA after 6 and 24 h incubation in culture medium with PBS (control) or 200 μg/mL IONPs, phase contrast, and fluorescent microscopy. White arrows indicate single cells with increased level of ROS production. Supplementary figure S5. Detection of late apoptotic/necrotic cells after IONPs treatment. SC-1 cells intravital staining with Nuclear Green after 6 and 24 h incubation in culture medium with PBS (control) or 200 μg/mL IONPs, phase contrast, and fluorescent microscopy. White arrows indicate rare dead cells. Supplementary figure S6. IONPs biodistribution profiles in multiple tumor models. Iron concentrations measured by AES in liver, spleen, kidney, lung, and heart of mice with 4T1 (a), CT26 (b), and B16 (c) tumors. Results are shown as means ± SEM. ∗ p < 0.05 in IONP-treated vs control group (Dunnett's Multiple Comparison Test). Supplementary figure S7. Patterns of MR contrast enhancing. T2-weighted imaging mode (left panels) was followed by T 2 ∗-weighted imaging (right panels). Representative images of diffuse (A, [file 8264208.f1.docx]

**
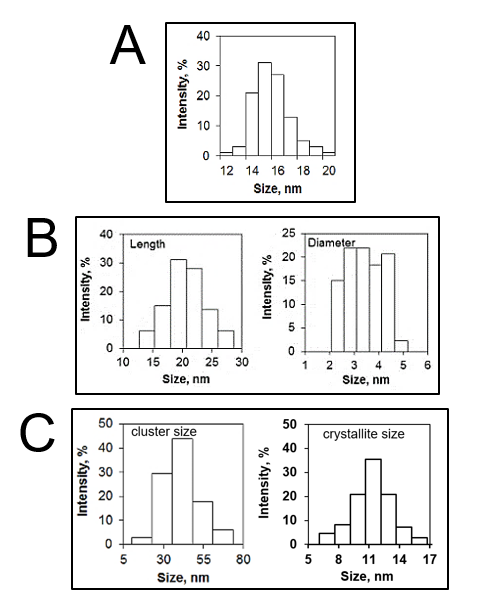
**

**Supplementary figure S1.** Histograms of MNCb **(A)**, MNRd **(B)** and MNCl **(C)** size distribution (associated with Fig 1A)


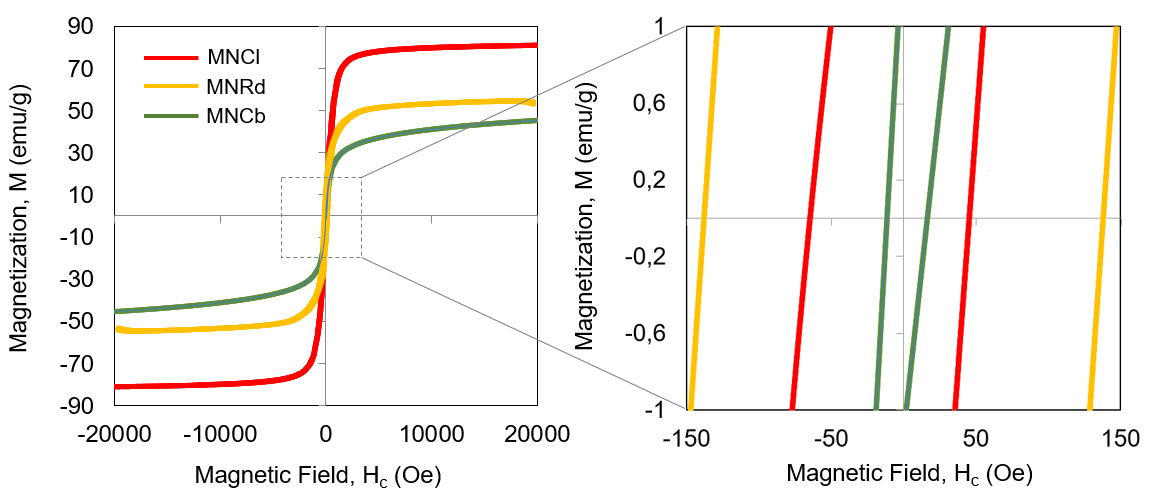


**Supplementary figure S2.** M-H curves of MNCb, MNRd and MNCl at room temperature. M-H hysteresis loops (from -20 to 20 kOe, 300 K) were obtained on «Quantum Design» Physical Property Measurement System (PPMS) equipped with vibration magnetometric device (VSM) with 2 mm amplitude of oscillations, 40 Hz frequency.


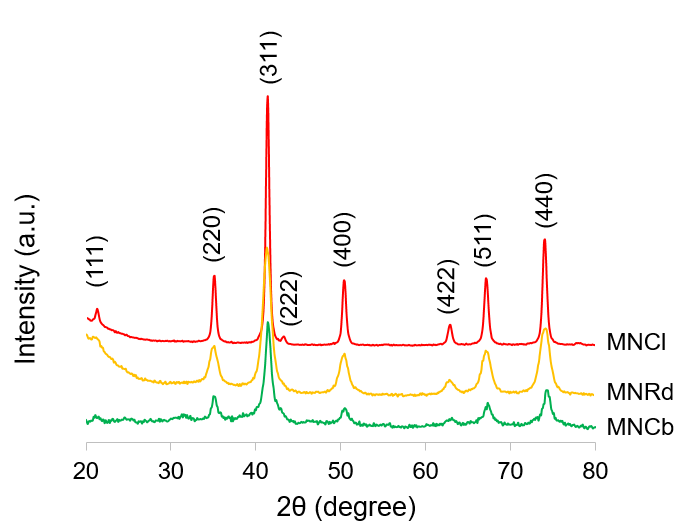


**Supplementary figure S3.** X-ray diffraction patterns of MNCb, MNRd and MNCl at room temperature. XRD patterns were obtained using an X-ray power diffractometer Rigaku Ultima IV with Co Kα radiation at room temperature. The data were collected from 2θ = 20 to 80° at a scan rate 0,1° per step and 3 s per point.


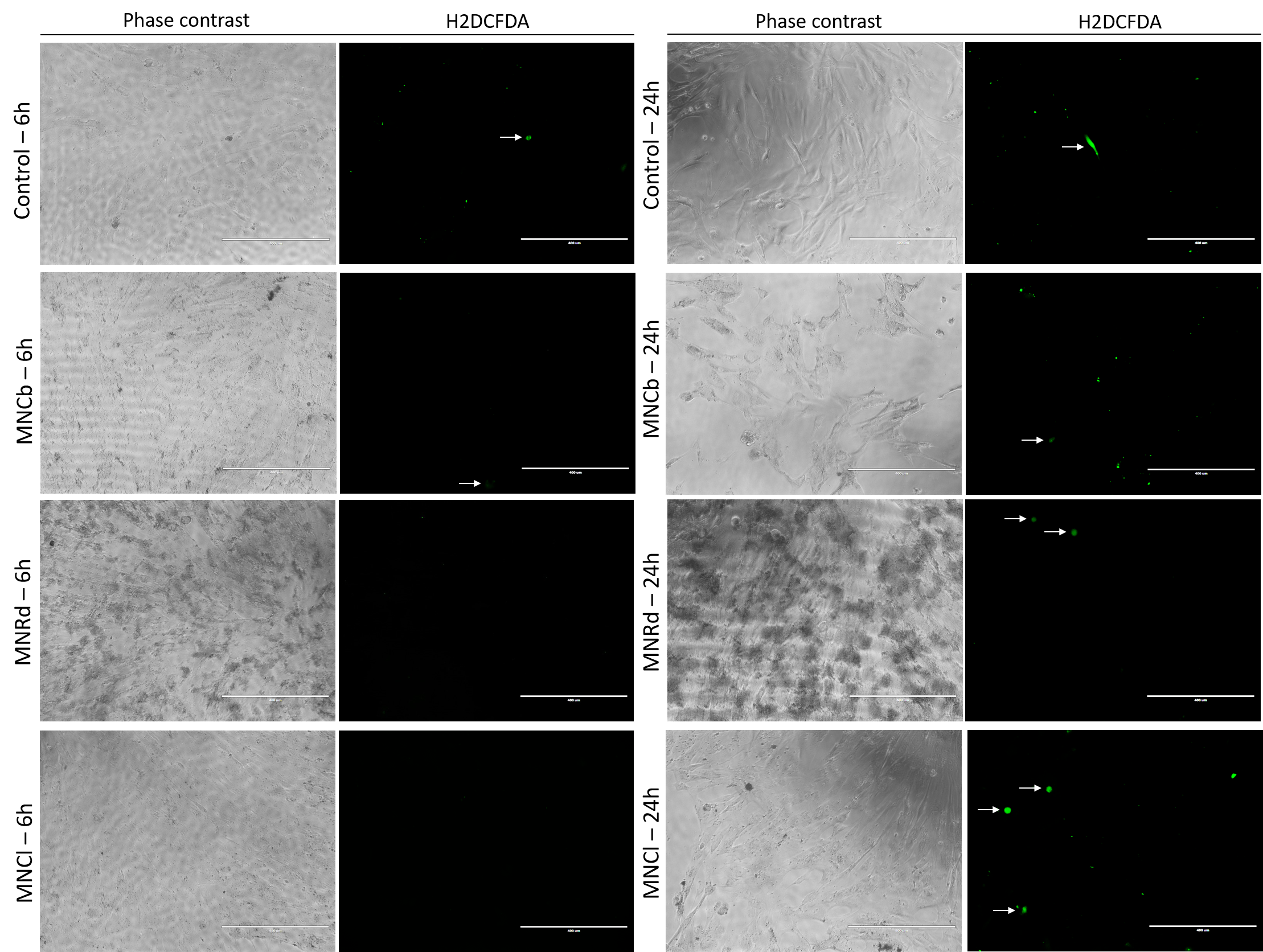


**Supplementary figure S4.** ROS detection in IONPs-treated cells. SC-1 cells intravital staining with H2DCFDA after 6 and 24 h incubation in culture medium with PBS (control) or 200 μg/mL IONPs, phase contrast and fluorescent microscopy. White arrows indicate single cells with increased level of ROS production.


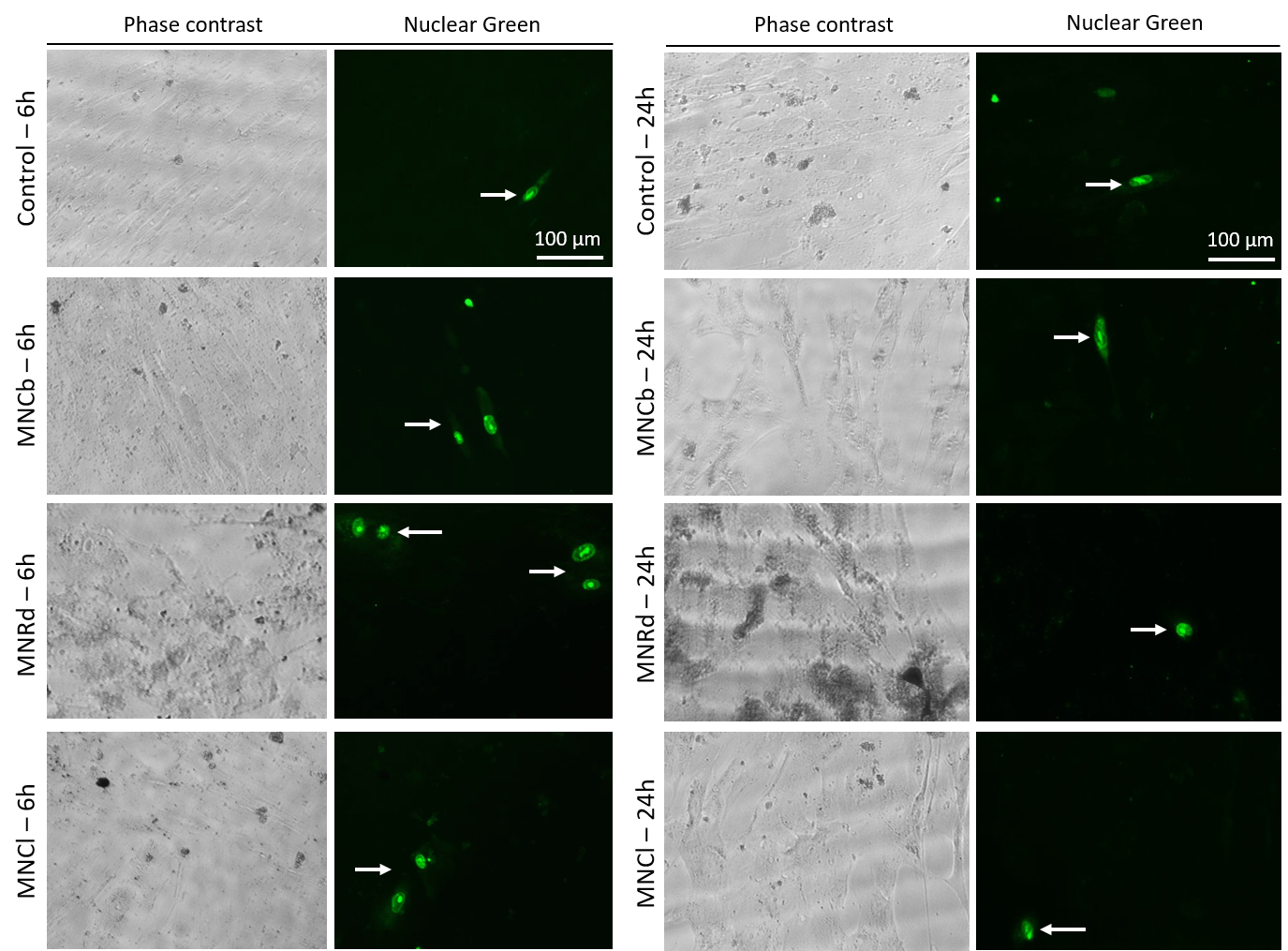


**Supplementary figure S5.** Detection of late apoptotic/necrotic cells after IONPs treatment. SC-1 cells intravital staining with Nuclear Green after 6 and 24 h incubation in culture medium with PBS (control) or 200 μg/mL IONPs, phase contrast and fluorescent microscopy. White arrows indicate rare dead cells.


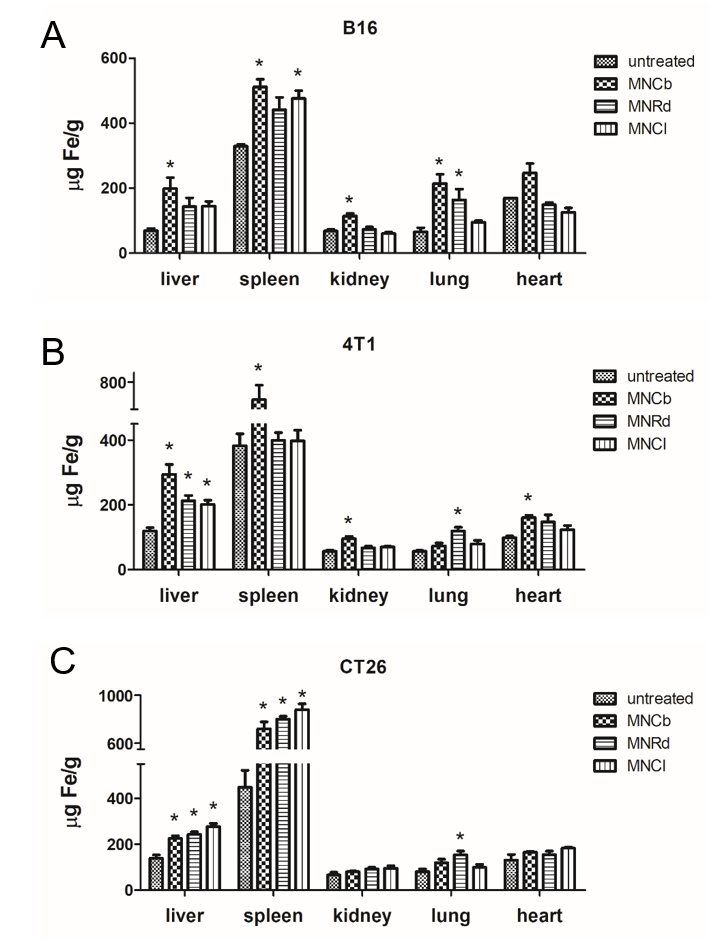


**Supplementary figure S6.** IONPs biodistribution profiles in multiple tumor models. Iron concentrations measured by AES in liver, spleen, kidney, lung and heart of mice with 4T1 (**A**), CT26 (**B**) and B16 (**C**) tumors. Results are shown as means ± SEM. *p<0.05 in IONP-treated vs control group (Dunnett's Multiple Comparison Test).

**
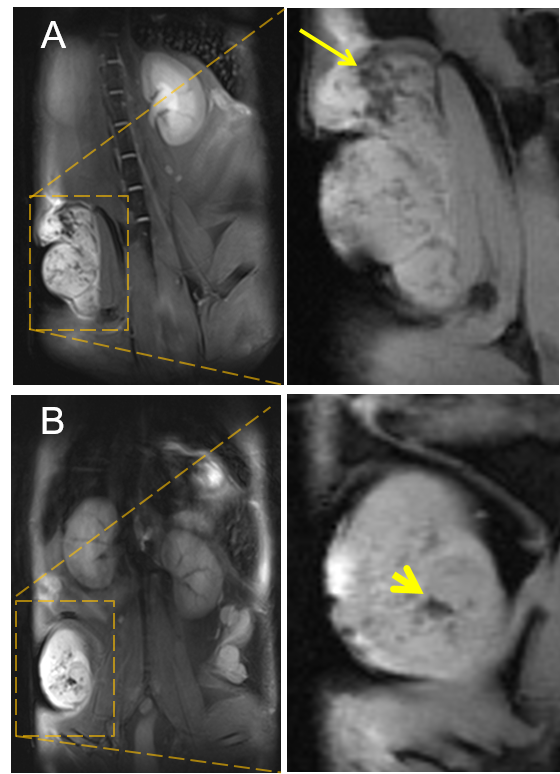
**

**Supplementary figure S7.** Patterns of MR contrast enhancing. T2-weighted imaging mode (left panels) was followed by T2*-weighted imaging (right panels). Representative images of diffuse (**A,** arrow) and focal (**B**, arrowhead) tumor contrast enhancement after intravenous injection of IONPs.

**Supplementary Table S1.** XRD characteristics of MNCb, MNRd and MNCl and standard magnetite nanoparticles.

| Sample | X-ray diffraction analysis | | |
| --- | --- | --- | --- |
|  | Volume fraction, % | Crystallite size, nm | Lattice constants, Å |
|  | Fe_3_O_4_ | Fe_3_O_4_ | Fe_3_O_4_ |
| MNCb | 100 | 14.1(3) | 8.390 |
| MNRd | 100 | 15.2(7) | 8.388 |
| MNCl | 100 | 18.4(3) | 8.392 |
| Standard magnetite | - | - | 8.396 |
